# A revised checklist of Pottiaceae (Bryophyta) in Mongolia

**DOI:** 10.3897/phytokeys.275.197551

**Published:** 2026-06-01

**Authors:** Enkhtaivan Enkhjargal, Qishuo Feng, Qin Qi, Dongping Zhao

**Affiliations:** 1 National University of Mongolia, Ulaanbaatar, 14201, Mongolia Inner Mongolia Engineering Technology Research Center of Germplasm Resources Conservation and Utilization, School of Life Sciences, Inner Mongolia University Hohhot China; 2 Sector of Plant Systematics, Botanic Garden and Research Institute, Mongolian Academy of Sciences, Ulaanbaatar 13270, Mongolia Key Laboratory of Herbage & Endemic Crop Biotechnology, Ministry of Education, School of Life Sciences, Inner Mongolia University Hohhot China; 3 Inner Mongolia Engineering Technology Research Center of Germplasm Resources Conservation and Utilization, School of Life Sciences, Inner Mongolia University, Hohhot 010020, China National University of Mongolia Ulaanbaatar Mongolia; 4 Key Laboratory of Herbage & Endemic Crop Biotechnology, Ministry of Education, School of Life Sciences, Inner Mongolia University, Hohhot 010020, China Sector of Plant Systematics, Botanic Garden and Research Institute, Mongolian Academy of Sciences Ulaanbaatar Mongolia

**Keywords:** Bryophyte, checklist, *
Didymodon
*, Mongolia, Pottiaceae

## Abstract

This updated checklist of Mongolian Pottiaceae is based on a thorough review of over 2,000 specimens from HIMC, UBA, and MO, along with published records. A total of 1,030 occurrence records were verified at the sum level, where “sum” refers to an administrative unit, identifying 99 taxa, including 93 species and six varieties in 26 genera. *Didymodon* is the most species-rich genus, with 27 species and two varieties. The update adds one genus and 20 taxa (16 species and four varieties) to the Mongolian flora, while excluding nine previously reported taxa. Three names remain doubtful pending voucher confirmation. For each accepted taxon, evidence and distribution details are provided at the province/city and sum levels, together with an identification key to the genera of Mongolian Pottiaceae. The geographic summaries also reveal strong documentation unevenness among provinces, with observed richness closely tracking collecting intensity. The checklist is therefore intended as a voucher-based national baseline and a guide for future gap-filling surveys.

## Introduction

Mongolia is a landlocked country in North Asia, with a strong continental climate, high temperature seasonality, and high variability in aridity and elevation. Its vegetation includes boreal and montane forest-steppe, steppe, and semi-desert to desert basins (Hilbig 1995). It has high mountains in the west and north, which contribute to habitat diversity and microrefugia. Rock outcrops, cliffs, gravel slopes, and open soils provide many microsites suitable for mosses adapted to exposed conditions.

Pottiaceae is one of the most species-rich and ecologically important families of mosses, and its members are frequent components of open, exposed, and seasonally dry habitats. The family has traditionally been treated as taxonomically difficult because many taxa are small, morphologically reduced, and strongly influenced by convergent adaptations to stressful environments (Zander 1993, 2006, 2007; Goffinet et al. 2009). Molecular studies have also shown that some traditionally recognized subfamilial and generic relationships within Pottiaceae are complex and require cautious interpretation (Werner et al. 2004; Inoue and Tsubota 2014). Many Pottiaceae species are desiccation-tolerant and can recover rapidly after rehydration, allowing them to occupy soil crusts, rock surfaces, gravel slopes, and disturbed open substrates. These traits are particularly relevant in Mongolia, where aridity, strong radiation, wind abrasion, and episodic water availability are major ecological filters. Recent work on dryland and biocrust-forming mosses further emphasizes that microhabitat conditions, soil stability, water pulses, and desiccation–rehydration cycles are central to the distribution and ecological roles of mosses in arid landscapes (Ladrón de Guevara et al. 2022).

Bryophyte exploration on the Mongolian Plateau began in the mid-19^th^ century with collections by foreign missionaries and experts, although early gatherings focused mainly on vascular plants and included only scattered moss specimens. Systematic study of Mongolian bryophytes started in the 1970s. Between 1976 and 1994, Abramova and Tsegmed conducted surveys in major mountain systems such as Khangai, Khentii, and Mongolian Altai, publishing foundational accounts (Abramova and Abramov 1976, 1978; Abramov and Abramova 1983; Abramova and Tsegmed 1983, 1987, 1989, 1994; Tsegmed 1988). Tsegmed (2001) provided the first national checklist with distribution data, listing 27 genera and 79 species of Pottiaceae. A targeted multinational expedition to the Gobi Altai in 2004 documented 44 Pottiaceae species in 17 genera (Ignatov et al. 2004). The most comprehensive national treatment remains the Moss Flora of Mongolia (Tsegmed et al. 2010), which recognized 24 genera and 81 taxa. Subsequent collecting and taxonomic work has added a new species and multiple national records (Müller 2009; Zhao et al. 2015, 2016, 2018; Wang et al. 2016; Surina et al. 2020; Wu et al. 2020; Wujisiguleng et al. 2020), indicating that the checklist remains dynamic and requires periodic, voucher-based updating under current nomenclatural and taxonomic concepts.

This checklist aims to provide an updated, voucher-based synthesis of the taxonomy and distribution of Mongolian Pottiaceae. More than 2,000 herbarium specimens were analyzed, and the literature was reviewed to link accepted names to reproducible distributions at both the province/city and sum levels, together with concise summaries of richness, occupancy, and collecting intensity. An identification key to the genera of Mongolian Pottiaceae is also provided. Because the available database reflects substantial documentary unevenness across administrative units, it is used here primarily to identify distributional patterns and survey gaps rather than to make formal province-level beta-diversity comparisons. Although distributional knowledge remains incomplete for some taxa and areas, this work represents a first step toward a modern, standardized treatment of Mongolian Pottiaceae and a baseline for targeted gap-filling surveys, integrative taxonomic refinement, and conservation-relevant biodiversity assessment.

## Materials and methods

Mongolia comprises one capital city, 21 provinces, and 330 sums. Analyses were conducted at both the province/city and sum levels. These administrative units provided a practical spatial framework for documenting occurrences and summarizing reproducible richness, occupancy, and documentary unevenness.

Subfamily and generic concepts follow the evolutionary morphological framework proposed by Zander (1993, 2006) and the treatment in Flora of North America (Zander 2007). In keeping with this morphology-based framework, the circumscription of Pottiaceae adopted here follows Zander throughout the checklist and generic key, and *Timmiella* is therefore retained in the family for the purposes of this treatment. Molecular phylogenetic studies have proposed alternative circumscriptions for some lineages, including the segregation of *Timmiella* into Timmiellaceae (Inoue and Tsubota 2014), but Zander’s framework is retained here to ensure consistency with the principal morphology-based floristic treatments used for specimen identification and checklist compilation. Terminology and circumscription have been verified against the Moss Flora of China (Li et al. 2001) and the Species Catalogue of China (Jia and He 2013). Within each subfamily, genera and species are listed alphabetically by scientific name.

Occurrence data were obtained from more than 2,000 specimens at the Herbarium of Inner Mongolia University (**HIMC**), the Herbarium of the Botanic Garden and Research Institute (**UBA**), and the Herbarium of the Missouri Botanical Garden (**MO**) and from published literature on Mongolian Pottiaceae. Records were included if they provided reliable taxonomic evidence, such as a voucher or published identification, and specified locality information to at least the province level. Records lacking locality details or reliable identification were excluded from quantitative summaries but considered in checklists. To ensure comparability across sources and avoid inflated incidence counts, administrative names (province/city and sum) were standardized using a controlled vocabulary, spelling variants and diacritics were corrected, and ambiguous cases were manually checked. Duplicate entries representing the same taxon in the same sum within a province/city, arising from multiple vouchers or repeated literature citations, were merged so that each accepted taxon contributed at most one sum-level occurrence per administrative unit. The final database used for quantitative analyses comprised 1,030 verified sum-level occurrence records.

Scientific names were standardized to accepted taxa by reconciling synonyms and misapplied names with the references mentioned above and evidence. To avoid artificially increasing species counts, a “species-unit” approach was used for quantitative analysis. If a province or sum had records of only one infraspecific taxon (variety) of a species, it was treated as the presence of the parent species for richness and occupancy calculations, not as a separate species. The infraspecific name was retained in the checklist and supplementary tables (Suppl. materials 1, 2) to maintain taxonomic detail.

**Figure 1.**
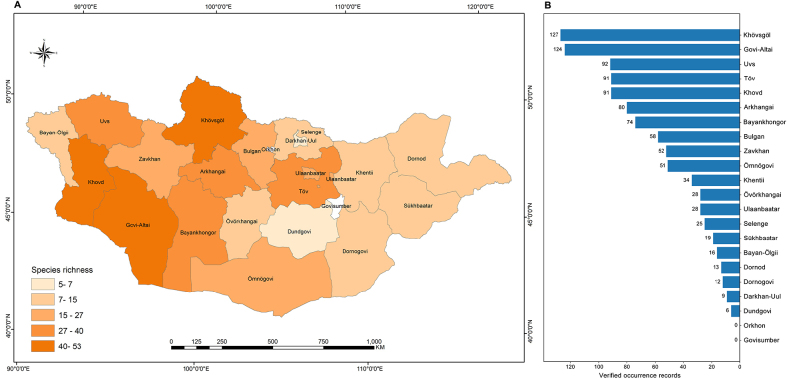
Observed species richness and sampling intensity of Mongolian Pottiaceae by province/city. **A**. Observed species richness; **B**. Sampling intensity, measured as the number of verified occurrence records.

**Figure 2.**
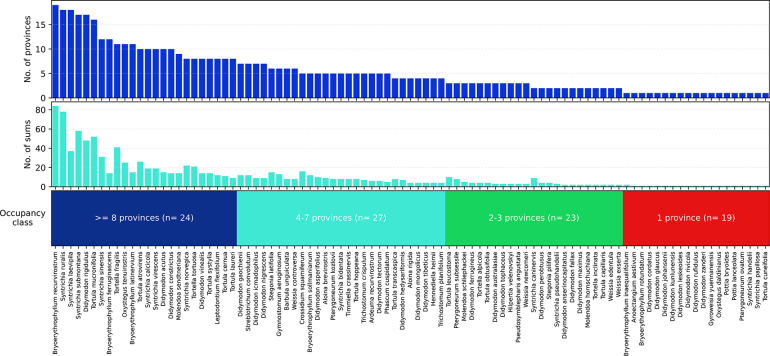
Species occupancy and rarity patterns of Mongolian Pottiaceae. Species (*x*-axis) are ordered by decreasing provincial occupancy, based on the number of provinces occupied. Top panel: number of provinces occupied per species. Middle panel: number of occupied sums per species. Bottom panel: occupancy classes, based on the number of provinces occupied (≥ 8, 4–7, 2–3, and 1 province), with the number of species in each class indicated (*n*).

**Figure 3.**
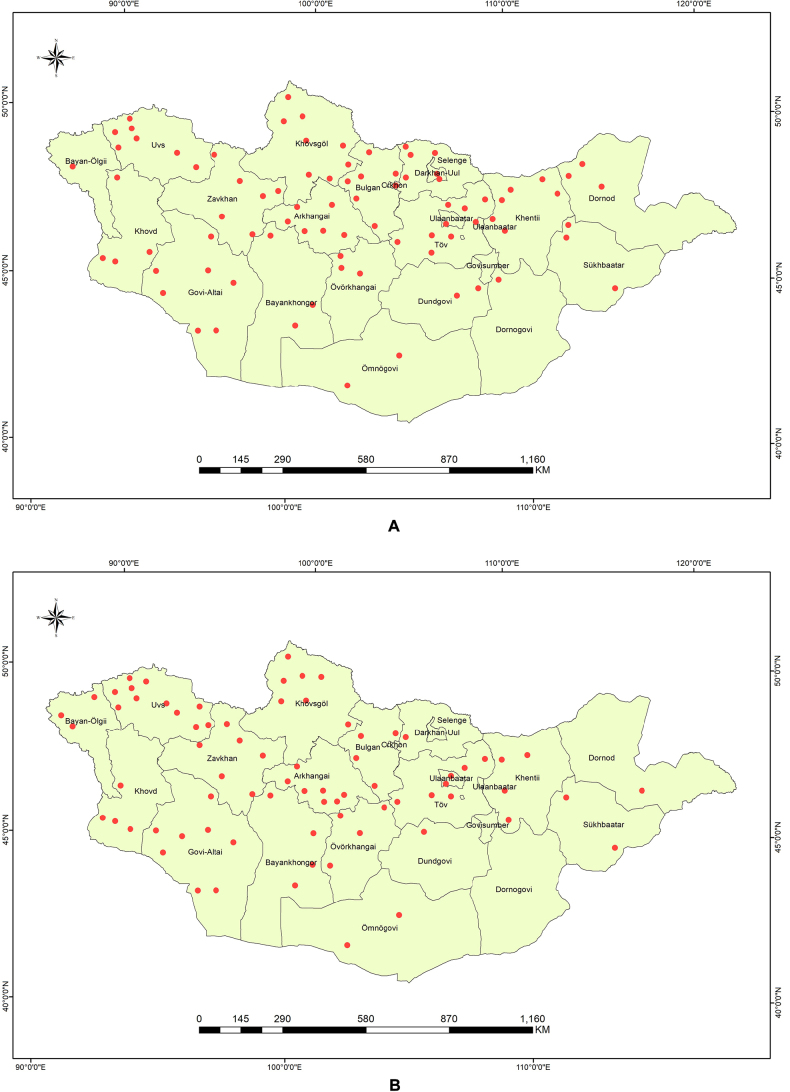
Sum-level distribution of two widespread Pottiaceae mosses in Mongolia. **A**. *Bryoerythrophyllum recurvirostrum*; **B**. *Syntrichia ruralis*.

The taxonomic composition was summarized by counting taxa per genus and subfamily (Suppl. material 1). Sampling intensity was quantified as the number of verified occurrence records per province/city and per sum (Suppl. material 2) and related to observed richness using Pearson correlation at the province/city level. Species occupancy and rarity patterns were quantified in Python 3.11.2 using pandas 2.2.3 (McKinney 2010) and NumPy 1.24.0 (Harris et al. 2020) by counting, for each species-unit, how many provinces/cities it occupied and how many sums it occupied after duplicate entries within the same province/city–sum combination were merged; species were then ranked by decreasing provincial occupancy and grouped into occupancy classes (≥8, 4–7, 2–3, and 1 province). Fig. 2 was prepared in Python 3.11.2 using Matplotlib 3.7.5 (Hunter 2007). The province/city-level maps of observed species richness and sampling intensity (Fig. 1) and the sum-level distribution maps of the two widespread species (Fig. 3) were prepared in ArcGIS 10.8.1. Because the database was designed as a voucher-anchored occurrence synthesis with duplicate taxon records merged within province/city–sum units, it was not used for formal province-level beta-diversity analyses. Instead, province-level summaries are interpreted primarily as indicators of documentary unevenness and priorities for future sampling.

## Results

### Checklist summary and taxonomic composition

Using the species-unit counting method, the revised checklist recognizes 99 accepted taxa of Pottiaceae in Mongolia, comprising 26 genera, 93 species, and six varieties (Suppl. material 1). The verified database used for quantitative summaries contains 1,030 sum-level occurrence records.

Taxonomic diversity is concentrated in a few large genera. *Didymodon* is the most species-rich genus (27 species and two varieties), representing 29.3% of all taxa. *Syntrichia* (12 species and one variety) and *Tortula* (11 species) represent the second most species-rich genera. *Bryoerythrophyllum* follows with six species, whereas the remaining 22 genera contain only one to five taxa each, showing a strongly long-tailed pattern typical of dryland Pottiaceae.

The 26 Mongolian genera are categorized into four subfamilies. Timmielloideae comprises one genus, *Timmiella*. Trichostomoideae includes five genera: *Oxystegus*, *Pseudosymblepharis*, *Tortella*, *Trichostomum*, and *Weissia*. Barbuloideae has 10 genera: *Anoectangium*, *Ardeuma*, *Barbula*, *Bryoerythrophyllum*, *Didymodon*, *Gymnostomum*, *Gyroweisia*, *Leptodontium*, *Molendoa*, and *Streblotrichum*. Pottioideae also has 10 genera: *Aloina*, *Crossidium*, *Hennediella*, *Hilpertia*, *Phascum*, *Pottia*, *Pterygoneurum*, *Stegonia*, *Syntrichia*, and *Tortula*.

### Geographic distribution, observed richness, and sampling intensity

The richest provinces are Khövsgöl, with 53 species; Govi-Altai, with 48 species; and Khovd, with 45 species, followed by Bayankhongor and Töv, with 40 and 36 species, respectively (Fig. 1A). Sampling intensity, measured as the number of verified occurrence records, was also highly uneven among provinces/cities (Fig. 1B). The most intensively documented provinces were Khövsgöl, Govi-Altai, Uvs, Khovd, and Töv, whereas Darkhan-Uul and Dundgovi were represented by very few verified records, and Orkhon and Govisumber had no verified sum-level records in the present database.

The observed richness and sampling intensity show that provinces with higher observed richness generally also have more verified occurrence records. This pattern was confirmed by a strong province/city-level correlation between sampling intensity and observed richness (Pearson’s *r* = 0.961), indicating that spatial variation in collecting intensity has likely exerted a major influence on the currently observed richness pattern.

At the sum level, richness is highly uneven: the median is 4.0 species per sum, with 46 sums (28.8%; *n* = 160) containing only one species (Suppl. material 2). The sums with the highest species richness are Renchinlkhümbe sum (Khövsgöl, 40 species), Bulgan sum (Khovd, 30 species), and Bogd sum (Bayankhongor, 30 species) (Table 1).

**Table 1.** Ten sums with the highest observed species richness.

No.	Sum	Province	Number of species
1	Renchinlkhümbe	Khövsgöl	40
2	Bulgan	Khovd	30
3	Bogd	Bayankhongor	30
4	Altanbulag	Töv	25
5	Ulaan-Uul	Khövsgöl	23
6	Khan-Uul District	Ulaanbaatar	23
7	Üyench	Khovd	22
8	Biger	Govi-Altai	19
9	Khaliun	Govi-Altai	19
10	Khankhongor	Ömnögovi	19

### Species occupancy and rarity

Occupancy distributions exhibit a right-skew across both provinces and sums (Fig. 2; Suppl. material 2). Nineteen species (20.4%) are found in only one province, and 23 species (24.7%) are found in two or three provinces. Only a small subset of taxa is widespread, occurring in eight or more provinces.

At the sum level, 18 species (19.4%) are found in only one sum. The most common species are *Bryoerythrophyllum recurvirostrum*, occurring in 20 provinces and 83 sums (Fig. 3A), and *Syntrichia ruralis*, occurring in 18 provinces and 76 sums (Fig. 3B), consistent with their broad ecological tolerance and frequent occurrence in open, disturbed, and drought-prone habitats.

### Documentary gaps and implications for future survey effort

The province-level summaries highlight marked documentary unevenness across Mongolia. Provinces with many verified records also show the highest observed richness, whereas provinces such as Darkhan-Uul and Dundgovi remain represented by very few records (Fig. 1A, B). This pattern, together with the strong record–richness correlation, indicates that current province-level richness values are still strongly influenced by collection history.

Because the present database is a deduplicated occurrence synthesis rather than a repeated collection-event dataset, it is best interpreted as a baseline for identifying survey gaps rather than as a basis for formal beta-diversity comparisons among provinces. Future work should reconstruct collection-event data and use species accumulation curves or other coverage-based approaches to assess how much additional sampling is needed to characterize the Pottiaceae flora of each province more fully.

### Checklist update dynamics

Checklist updating and critical reassessment yielded one new genus record for Mongolia, 16 newly recorded species, and four newly recorded varieties (Suppl. material 1). Additionally, nine previously reported taxa were identified for exclusion from the Mongolian flora, and three doubtful names were flagged pending voucher confirmation.

### New genus records

***Pseudosymblepharis* Broth**.

### New species records

**1. *Anoectangium aestivum* (Hedw.) Mitt**.

**Specimens examined. Mongolia • Arkhangai Province**: Khairkhan Sum, T.R. Zhang ZM201508082 (HIMC).

**2. *Bryoerythrophyllum inaequalifolium* (Taylor) R.H. Zander**

**Specimens examined. Mongolia • Bulgan Province**: Gurvanbulag Sum, Ts. Tsegmed 12129; Khangal Sum, Ts. Tsegmed 11572 (UBA).

**3. *Bryoerythrophyllum rotundatum* (Lindb. & Arnell) P.C. Chen**

**Specimens examined. Mongolia • Bayankhongor Province**: Bogd Sum, Ts. Tsegmed & B. Munkhjargal 12571, 12777 (UBA).

**4. *Bryoerythrophyllum sollmanianum* J.A. Jiménez, M.J. Cano & Shevock**

**Specimens examined. Mongolia • Arkhangai Province**: Erdenemandal Sum, Ts. Tsegmed & B. Amar 1888, 1890, 1895, 1904; Tsetserleg Sum, N. Ulzikhutag 432; Tüvshrüülekh Sum, Ts. Tsegmed & B. Amar 5003, Ts. Tsegmed 5410, Ts. Tsegmed & A.Baatar 4975; • **Bulgan Province**: Gurvanbulag Sum, Ts. Tsegmed 2923; Khangal Sum, Ts. Tsegmed 10648; Khutag-Öndör Sum, Ts. Tsegmed 2650; • **Khövsgöl Province**: Renchinlkhümbe Sum, E. Enkhjargal 2023; • **Töv Province**: Altanbulag Sum, Ts. Tsegmed & L. Darambazar 13159, 13167; Erdene Sum, N.S. Golubkova & U. Tsogt 28, Ts. Tsegmed & A. Baatar 10084; Möngönmorit Sum, Ts. Tsegmed 9519, 9520; Sergelen Sum, Ts. Tsegmed 11896; • **Ulaanbaatar City**: Khan-Uul District, Ts. Tsegmed 2427, 2431(UBA).

**5. *Didymodon rivicola* (Broth.) R.H. Zander**

**Specimens examined. Mongolia • Govi-Altai Province**: Altai Sum, Ts. Tsegmed 12824 (UBA).

**6. *Molendoa hornschuchiana* (Hook.) Lindb. ex Limpr**.

**Specimens examined. Mongolia • Bayankhongor Province**: Bogd Sum, Ts. Tsegmed & B. Munkhjargal 12777 (UBA); • **Uvs Province**: Tarialan Sum, Ts. Tsegmed 3844 (UBA).

**7. *Oxystegus daldinianus* (De Not.) Köckinger, O. Werner & Ros**

**Specimens examined. Mongolia • Töv Province**: Erdene Sum, Ts. Tsegmed 10270 (UBA).

**8. Phascum cuspidatum var. schreberianum (Dicks.) Brid**.

**Specimens examined. Mongolia • Arkhangai Province**: Tüvshrüülekh Sum, Ts. Tsegmed 5075 (UBA); • **Bayankhongor Province**: Bogd Sum, Ts. Tsegmed & B. Munkhjargal 12574 (UBA); • **Khövsgöl Province**: Renchinlkhümbe Sum, E. Enkhjargal 148, 170 (UBA); • **Ulaanbaatar City**: Khan-Uul District, Ts. Tsegmed 897 (UBA); D.P. Zhao 201308122, 201308129, 201308150, 201308151 (HIMC); • **Övörkhangai Province**: Bayan-Öndör Sum, Ts. Tsegmed 8236 (UBA).

**9. *Pseudosymblepharis angustata* (Mitt.) Hilp**.

**Specimens examined. Mongolia • Khovd Province**: Üyench Sum, Ts. Tsegmed 9136, 9192 (UBA); • **Khövsgöl Province**: Renchinlkhümbe Sum, E. Enkhjargal 528, 531, 782, 1799, 1810 (UBA); • **Töv Province**: Erdene Sum, N.S. Golubkova & U. Tsogt 61 (UBA).

**10. *Stegonia pilifera* (Brid.) H.A. Crum & L.E. Anderson**.

**Specimens examined. Mongolia • Khovd Province**: Bulgan Sum, Ts. Tsegmed 8996; • **Uvs Province**: Bökhmörön Sum, Ts. Tsegmed 4296; Sagil Sum, Ts. Tsegmed 3983; Tarialan Sum, Ts. Tsegmed 4802 (UBA).

**11. *Syntrichia calcicola* J.J. Amann**

**Specimens examined. Mongolia • Arkhangai Province**: Bulgan Sum, N.S. Golubkova & U. Tsogt 195, 254 (UBA); Tariat Sum, Ts. Tsegmed 3036 (UBA); Tsetserleg Sum, Ts. Tsegmed 12113 (UBA); Tüvshrüülekh Sum, Ts. Tsegmed 5465 (UBA); • **Bayankhongor Province**: Bogd Sum, Ts. Tsegmed & E. Enkhjargal 12730 (UBA); • **Bulgan Province**: Gurvanbulag Sum, Ts. Tsegmed 2791, 2842 (UBA); • **Khentii Province**: Batshireet Sum, N.S. Golubkova & U. Tsogt 21 (UBA); • **Khovd Province**: Bulgan Sum, Ts. Tsegmed 8689, 8740 (UBA); • **Khövsgöl Province**: Renchinlkhümbe Sum, Ts. Tsegmed 114, 377 (UBA); E. Enkhjargal & C. Montagne 678 (UBA), E. Enkhjargal 121, 130, 256, 1736 (UBA); Tsagaannuur Sum, E. Enkhjargal 2902, 2910 (UBA); Ulaan-Uul Sum, E. Enkhjargal 2078, 2300, 2405 (UBA); • **Töv Province**: Altanbulag Sum, Ts. Tsegmed 13220, 13351, 13355 (UBA); Bornuur Sum, Ts. Tsegmed 892 (UBA); Sergelen Sum, Ts. Tsegmed 11766, 11787 (UBA); • **Ulaanbaatar City**: Khan-Uul District, Ts. Tsegmed 11951 (UBA); • **Uvs Province**: Baruunturuun Sum, Ts. Tsegmed 1335 (UBA); Tarialan Sum, Ts. Tsegmed 3691, 3733 (UBA); • **Zavkhan Province**: Otgon Sum, Ts. Tsegmed & B. Amar 1013 (UBA); Tosontsengel Sum, Ts. Tsegmed 5717 (UBA).

**12. Syntrichia caninervis var. gypsophila (J.J. Amann ex G. Roth) Ochyra**

**Specimens examined. Mongolia • Govi-Altai Province**: Bugat Sum, Ts. Tsegmed 8378, 8387 (UBA); • **Khovd Province**: Bulgan Sum, Ts. Tsegmed 8867, 8890, 8952 (UBA).

**13. *Syntrichia handelii* (Schiffn.) S. Agnew & Vondr**.

**Specimens examined. Mongolia • Govi-Altai Province**: Altai Sum, Ts. Tsegmed & B. Munkhjargal 12829, 12830 (UBA).

**14. *Syntrichia pseudohandelii* (J. Froehl.) S. Agnew & Vondr**.

**Specimens examined. Mongolia • Govi-Altai Province**: Bugat Sum, Ts. Tsegmed 8380 (UBA); Khaliun Sum, Ts. Tsegmed 8344; • **Khovd Province**: Bulgan Sum, Ts. Tsegmed 9023 (UBA).

**15. *Timmiella crassinervis* (Hampe) L.F. Koch**

**Specimens examined. Mongolia • Arkhangai Province**: Tüvshrüülekh Sum, Ts. Tsegmed 5237 (UBA); • **Bulgan Province**: Khangal Sum, Ts. Tsegmed 10842, 11567, 11613, 11617 (UBA); Khutag-Öndör Sum, Ts. Tsegmed 1770, 1803, 2464, 2655 (UBA); Teshig Sum, Ts. Tsegmed 10943 (UBA); • **Khövsgöl Province**: Tarialan Sum, Ts. Tsegmed 11132 (UBA); • **Selenge Province**: Baruunbüren Sum, Ts. Tsegmed 4584, 7591 (UBA); Tüshig Sum, Ts. Tsegmed 2192, 2193 (UBA); • **Zavkhan Province**: Tosontsengel Sum, Ts. Tsegmed 5716, 5792 (UBA).

**16. Tortella inclinata var. densa (Lorentz & Molendo) Limpr**.

**Specimens examined. Mongolia • Bayankhongor Province**: Galuut Sum, A. Rasuna 29500 (UBA); • **Khövsgöl Province**: Renchinlkhümbe Sum, E. Enkhjargal 778, 1477 (UBA).

**17. Tortella tortuosa var. fragilifolia (Jur.) Limpr**.

**Specimens examined. Mongolia • Arkhangai Province**: Tariat Sum, Ts. Tsegmed 3124 (UBA); Tüvshrüülekh Sum, Ts. Tsegmed 5070 (UBA); • **Khovd Province**: Üyench Sum, Ts. Tsegmed 9192 (UBA); • **Khövsgöl Province**: Renchinlkhümbe Sum, E. Enkhjargal 252 (UBA).

**18. *Tortula capillaris* (Dixon ex P.C. Chen) R.H. Zander**

**Specimens examined. Mongolia • Bayan-Ölgii Province**: Nogoonnuur Sum, Ts. Tsegmed 4382; • **Khovd Province**: Erdenebüren Sum, N. Ulzikhutag 187 (UBA).

**19. *Weissia edentula* Mitt**.

**Specimens examined. Mongolia • Khentii Province**: Batshireet Sum, Ts. Tsegmed sn.; • **Khövsgöl Province**: Renchinlkhümbe Sum, E. Enkhjargal 1064 (UBA).

**20. *Weissia newcomeri* (E.B. Bartram) K. Saito**.

**Specimens examined. Mongolia • Govi-Altai Province**: Altai Sum, N.S. Golubkova & U. Tsogt 64; • **Khentii Province**: Batshireet Sum, N.S. Golubkova & U. Tsogt b425; • **Uvs Province**: Bökhmörön Sum, Ts. Tsegmed sn. (UBA).

### Excluded species

1. *Bryoerythrophyllum alpigenum* (Venturi ex Jur.) P.C. Chen

2. *Bryoerythrophyllum rubrum* (Jur. ex Geh.) P.C. Chen

3. *Pseudocrossidium revolutum* (Brid.) R.H. Zander

4. *Weissia brachycarpa* (Nees & Hornsch.) Jur.

5. *Weissia condensa* (Voit) Lindb.

6. *Syntrichia princeps* (De Not.) Mitt.

7. *Syntrichia substereidosa* (Kramer) Tsegmed

8. *Timmiella anomala* (Bruch & Schimp.) Limpr

9. *Trichostomum arcticum* Kaal.

### Doubtful species

*Pseudocrossidium obtusulum* (Lindb.) H.A. Crum & L.E. Anderson

*Didymodon luridus* Hornsch.

*Syntrichia papillosissima* (Copp.) Loeske

## Discussion

This checklist updates the most recent national monograph and consolidates dispersed subsequent records into a single, reproducible baseline (Suppl. materials 1, 2). The resulting taxonomic structure is strongly long-tailed: a few genera contain most of the diversity, whereas many genera are represented by only one to a few taxa. This pattern is consistent with the broader ecology and taxonomy of Pottiaceae in dryland and cold-region habitats, where a limited number of lineages, notably *Didymodon*, *Syntrichia*, and *Tortula*, are especially successful in heterogeneous open microsites, whereas many other genera are more localized or sporadically collected (Zander 1993, 2007; Goffinet et al. 2009; Ladrón de Guevara et al. 2022).

*Didymodon* is the most diverse genus of Pottiaceae in Mongolia. Similar patterns exist in many continental drylands where *Didymodon* and related lineages exploit soil- and rock-associated microhabitats (Zander 1993, 2007). At the same time, *Didymodon* remains taxonomically challenging because morphological convergence and environmentally induced plasticity can obscure species boundaries, especially in regions with episodic wetting–drying cycles, wind abrasion, and high insolation (Zander 1993, 2006; Li et al. 2001; Werner et al. 2004). The voucher-based approach reduces uncertainty by anchoring records to specimens and applying current generic and subfamilial concepts; nevertheless, continued integrative revision will be necessary for critical complexes and sparsely represented taxa.

Geographic summaries show large spatial differences between observed species richness and sampling effort. The close correlation between record count and observed richness indicates that sampling bias has strongly affected the apparent spatial patterns. This is consistent with the fragmented history of bryological exploration in Mongolia and the tendency of records to be concentrated along expedition routes and in well-studied regions (Tsegmed 2001; Ignatov et al. 2004; Müller 2009; Tsegmed et al. 2010). Provinces with very few records, as well as many sums with only one species, are likely undersurveyed and should be prioritized in future gap-filling surveys.

Occupancy distributions are notably right-skewed at both the province and sum levels, with many taxa found in only a few administrative units. In specimen-based datasets, low occupancy requires careful interpretation. A taxon recorded in only one province or sum may indicate true ecological restriction, but it could also result from limited collection effort or challenges in detecting or identifying small, ephemeral, or morphologically variable taxa. Thus, the value of the checklist lies not only in identifying rarity but also in setting a prioritized agenda for validation. Taxa recorded from a single administrative unit, supported by clear diagnostic features and multiple collections from the same locality, should be prioritized for targeted recollection in similar habitats. Conversely, taxa with few collections, ambiguous localities, or those in taxonomically challenging groups should be re-examined carefully, with additional sampling to capture within-taxon variation. In this context, the widespread presence of *Bryoerythrophyllum recurvirostrum* and *Syntrichia ruralis* across many provinces and sums is expected. This reflects their broad ecological tolerance and frequent occupation of exposed, drought-prone substrates. Their extensive sum-level distributions also serve as an internal check on the consistency of administrative-unit standardization and data harmonization.

Although the present dataset does not justify formal province-level compositional comparisons, it still allows cautious ecological interpretation of broad geographic patterns in Mongolian Pottiaceae. At a coarse scale, the observed patterns are consistent with Mongolia’s major environmental gradients (Hilbig 1995; Dulamsuren et al. 2005; Mühlenberg et al. 2012). Northern and central mountainous regions, especially Khövsgöl, parts of Khangai, and adjacent forest-steppe areas, are likely to support relatively high Pottiaceae richness because they combine elevational heterogeneity, contrasting slope exposures, rocky substrates, open soil patches, and locally buffered moisture conditions. Such combinations increase the availability of microsites for both xerophytic and montane taxa and may help explain why several well-sampled sums in these regions rank among the richest administrative units in the present dataset.

By contrast, the Gobi and drier steppe regions probably favor a different subset of Pottiaceae adapted to strong aridity, high insolation, wind abrasion, and episodic water availability. In these landscapes, diversity may be structured less by closed vegetation types than by the fine-scale availability of exposed soil, gravel, rock crevices, and temporary moisture-retaining microsites (Zander 1993; Ignatov et al. 2004). The western mountains, including the Mongolian Altai and adjacent areas, are also likely to be important because steep elevational gradients and complex topography compress multiple habitat types within relatively small areas, potentially promoting local coexistence of cold-desert, steppe, and montane elements. In this sense, the checklist points not only to geographic gaps in collecting, but also to a broader environmental mosaic that is likely to underlie Pottiaceae diversity across Mongolia.

The present database was assembled to produce a reproducible national checklist and administrative-unit occurrence synthesis, rather than to estimate fully sampled provincial floras. These biogeographic interpretations should therefore be treated as working hypotheses rather than definitive province-level conclusions. Because the intensity of collecting remains highly uneven among provinces and sums, some apparent spatial contrasts may still reflect documentary bias rather than true floristic structure. Even so, the current checklist provides a useful framework for targeting future surveys across the main environmental domains of Mongolia, including northern forest-steppe mountains, western high-mountain systems, and the dry steppe-to-desert transition belt. Future work should reconstruct collection-event data and apply species accumulation curves or other coverage-based assessments to determine how much additional sampling is needed in each province and to test whether the broad patterns suggested here are robust (Chao and Jost 2012).

The checklist revision indicates that knowledge of Mongolian Pottiaceae remains dynamic as additional collecting and taxonomic work continue. Tsegmed et al. (2010) reported 24 genera and 81 taxa in the most recent comprehensive monograph, whereas this voucher-based revision recognizes 26 genera and 99 taxa, including one newly recorded genus and 20 newly recorded taxa. A critical review of published records and specimen vouchers also resulted in the exclusion of nine previously reported taxa, while three names remain doubtful pending voucher confirmation. Together, the revised checklist and the accompanying occurrence summaries provide a starting point for future targeted field surveys, integrative taxonomic revisions, biodiversity inventories, and conservation-relevant assessment in Mongolia and adjacent regions of Central Asia.

### Key to the genera of Pottiaceae in Mongolia

**Table:** 

1	Upper lamina bistratose, except at margins; the two layers slightly alternating near the costa and becoming vertically aligned towards the margins; leaves elliptic to broadly lanceolate (subfam. Timmielloideae)	*** Timmiella ***
–	Upper laminal cells usually unistratose; if bistratose, then layers aligned vertically; leaves ovate to lanceolate	**2**
2	Leaves lanceolate to linear, incurved, commonly tubulose, and often contorted or spiraled when dry; margins plane to incurved; upper laminal KOH color reaction yellow (occasionally orange); costa lacking distinct differentiated dorsal epidermis (similar to stereids); stem hyalodermis usually present, sclerodermis weakly differentiated (subfam. Trichostomoideae)	**3**
–	Leaves lanceolate, ovate, lingulate or spathulate; when dry, leaves tightly appressed to stem or slightly contorted; margins recurved to revolute, rarely plane; upper laminal KOH color reaction yellow to red; costal dorsal epidermis differentiated or not; stem hyalodermis variously differentiated, hyalodermis differentiated or absent	**7**
3	Leaf margins involuted, rarely incurved; monoicous	*** Weissia ***
–	Leaf margins plane; dioicous	**4**
4	Basal laminal hyaline cells forming a distinct V-shaped pattern extending up from the leaf base	*** Tortella ***
–	Basal laminal hyaline cells forming a straight or U-shaped band at the leaf base	**5**
5	Leaves undulate and fragile, margins often breaking into notch-like incisions; upper leaf margins entire or with irregular small rounded teeth formed by projecting cell walls and papillae	*** Oxystegus ***
–	Leaves flat and not fragile; upper leaf margins entire or with regular fine teeth formed by cell papillae	**6**
6	Leaf base sheathing; basal hyaline cells extending up along margins in a U-shape; at leaf base, costal ventral stereid band with more than or equal layers compared to the dorsal one	*** Pseudosymblepharis ***
–	Leaf base not sheathing; basal hyaline cells not extending upwards, forming a straight basal hyaline band; at leaf base, costal ventral stereid band with fewer or equal layers compared to the dorsal one	*** Trichostomum ***
7	Leaves broadly lanceolate to narrowly elliptic, occasionally broadly lingulate; costa usually with both dorsal and ventral stereid bands (except in *Anoectangium* and *Gyroweisia*); gemmae present, usually clavate; stem sclerodermis differentiated (subfam. Barbuloideae)	**8**
–	Leaves usually broadly lingulate to spathulate; costa with only a dorsal stereid band; rod-like gemmae uncommon; stem thick-walled cortical layer absent or only weakly developed (subfam. Pottioideae)	**17**
8	Perichaetia on lateral branches	**9**
–	Perichaetia terminal on main stem	**10**
9	Leaves strongly keeled; costa with dorsal stereid band only	*** Anoectangium ***
–	Leaves not strongly keeled; costa with dorsal and ventral stereid bands	*** Molendoa ***
10	Costa with dorsal stereids only; annulus of 2–3 rows of highly vesiculose cells	*** Gyroweisia ***
–	Costa with dorsal and ventral stereids; annulus slightly differentiated or of 1–3 rows of vesiculose cells	**11**
11	Stem central strand absent; costal epidermis not differentiated	**12**
–	Stem central strand present; costal epidermis usually differentiated	**13**
12	Stem hyalodermis absent; leaf margins entire, plane above; axillary gemmae absent	*** Ardeuma ***
–	Stem hyalodermis present; upper margins often toothed; axillary gemmae usually present	*** Leptodontium ***
13	Axillary hairs with a basal brown cell; laminal cells smooth or with sparse, low papillae	*** Didymodon ***
–	Axillary hairs all hyaline; laminal cells with dense low papillae or bifurcate papillae	**14**
14	Plants greenish-grey above, pale brown below; laminal cells with single or bifurcate solid papillae, often crowded and obscuring cell outlines; costa percurrent or ending 2–5 cells below the apex; peristome absent	*** Gymnostomum ***
–	Plants bright green or yellow-brown above, yellow or reddish-brown below; laminal cells with bifurcate to multifurcate hollow papillae; costa percurrent or slightly excurrent; peristome usually present	**15**
15	Upper laminal KOH color reaction red; cells on ventral surface of upper costa quadrate; leaf apex or upper margins often toothed	*** Bryoerythrophyllum ***
–	Upper laminal KOH color reaction yellow; cells on ventral surface of upper costa elongate; leaf apex and upper margins usually entire	**16**
16	Gemmae absent	*** Barbula ***
–	Gemmae often present in leaf axils or on rhizoids	*** Streblotrichum ***
17	Ventral leaf surface with lamellae or filaments	**18**
–	Ventral leaf surface without lamellae or filaments	**20**
18	Costal ventral surface with 2–4 rows of lamellae	*** Pterygoneurum ***
–	Ventral leaf surface with numerous filaments on the lamina and costa	**19**
19	Filaments on lamina and costa; leaf margins incurved; costa percurrent or excurrent as reddish-brown awn	*** Aloina ***
–	Filaments restricted to costa; leaf margins recurved or revolute; costa excurrent as hyaline hair-point	*** Crossidium ***
20	Leaf margins strongly revolute, forming a spiral tube	*** Hilpertia ***
–	Leaf margins plane to recurved, marginal cells without the above specialisation	**21**
21	Plants bulbiform; leaves broadly ovate to nearly round, imbricate	*** Stegonia ***
–	Plants usually elongate; leaves not imbricate	**22**
22	Capsule immersed, cleistocarpous	*** Phascum ***
–	Capsule exserted, dehiscent	**23**
23	Peristome absent or rudimentary	**24**
–	Peristome present, linear or filiform	**25**
24	Upper margins serrulate; border of 2–4 rows of elongate smooth cells differentiated	*** Hennediella ***
–	Upper margins entire or with papillose denticulation; border not differentiated	*** Pottia ***
25	Basal hyaline cells forming an M-shaped pattern; costa excurrent as long hair-point; peristome basal membrane high and tubular; upper laminal KOH reaction red	*** Syntrichia ***
–	Basal hyaline cells forming a straight band; costa excurrent as short apiculus; peristome basal membrane low; upper laminal KOH reaction yellow	*** Tortula ***

## Additional information

### Artificial Intelligence (AI) use

The authors accept full responsibility for the content of the manuscript, including the disclosure of any use of AI.

No AI tools were used in the preparation of this manuscript.

### Author ORCIDs

E. Enkhjargal https://orcid.org/0000-0003-2056-0497

D. Zhao https://orcid.org/0000-0002-0031-7003

### Data availability

All of the data that support the findings of this study are available in the main text or Supplementary Information.

## Supplementary materials

Supplementary material 1 Full checklist of Mongolian Pottiaceae Data type xlsx Explanation note Voucher-anchored checklist of all accepted taxa, organised by subfamily and genus, with standardised names, evidence and distribution at province/city and sum levels. The file also lists newly-recorded taxa, excluded taxa and doubtful names pending confirmation. https://binary.pensoft.net/file/1665216This dataset is made available under the Open Database License (http://opendatacommons.org/licenses/odbl/1.0/). The Open Database License (ODbL) is a license agreement intended to allow users to freely share, modify, and use this Dataset while maintaining this same freedom for others, provided that the original source and author(s) are credited. Enkhtaivan Enkhjargal, Qishuo Feng, Qin Qi, Dongping Zhao

Supplementary material 2 Geographic distribution and occupancy summaries Data type xlsx Explanation note Province/city- and sum-level summaries of sampling intensity (verified occurrence record counts), observed richness and occupancy of each species-unit (numbers of occupied province/city units and sums), including summaries of rarity classes, the richest administrative units and province-level contrasts that help identify documentary gaps and priorities for future surveys. https://binary.pensoft.net/file/1665217This dataset is made available under the Open Database License (http://opendatacommons.org/licenses/odbl/1.0/). The Open Database License (ODbL) is a license agreement intended to allow users to freely share, modify, and use this Dataset while maintaining this same freedom for others, provided that the original source and author(s) are credited. Enkhtaivan Enkhjargal, Qishuo Feng, Qin Qi, Dongping Zhao
